# Comparison of quantitative real time PCR with Sequencing and ribosomal RNA-FISH for the identification of fungi in Formalin fixed, paraffin-embedded tissue specimens

**DOI:** 10.1186/1471-2334-11-202

**Published:** 2011-07-26

**Authors:** Volker Rickerts, Prasanna D Khot, David Myerson, Daisy L Ko, Evelyn Lambrecht, David N Fredricks

**Affiliations:** 1 Department of Internal Medicine II, University hospital, Frankfurt, Germany; 2 Fred Hutchinson Cancer Research Center, Seattle, WA, USA; 3 Department for Pathology, University hospital, Frankfurt, Germany

## Abstract

**Background:**

Identification of the causative agents of invasive fungal infections (IFI) is critical for guiding antifungal therapy. Cultures remain negative in a substantial number of IFI cases. Accordingly, species identification from formalin fixed, paraffin embedded (FFPE) tissue specimens by molecular methods such as fluorescence in situ hybridisation (FISH) and PCR provides an appealing approach to improve management of patients.

**Methods:**

We designed FISH probes targeting the 28S rRNA of Aspergillus and Candida and evaluated them with type strains. Fluorescence microscopy (FM), using FISH probes and quantitative broad-range fungal PCR targeting the rRNA gene were applied to FFPE tissue specimens from patients with proven IFI in order to explore benefits and limitations of each approach.

**Results:**

PCR followed by sequencing identified a broad spectrum of pathogenic fungi in 28 of 40 evaluable samples (70%). Hybridisation of FISH probes to fungal rRNA was documented in 19 of 40 tissue samples (47.5%), including 3 PCR negative samples with low fungal burden. The use of FISH was highly sensitive in invasive yeast infections, but less sensitive for moulds. In samples with hyphal elements, the evaluation of hybridisation was impaired due to autofluorescence of hyphae and necrotic tissue background.

**Conclusions:**

While PCR appears to be more sensitive in identifying the causative agents of IFI, some PCR negative and FISH positive samples suggest that FISH has some potential in the rapid identification of fungi from FFPE tissue samples.

## Background

The identification of the causative agents of invasive fungal infections (IFI) is critical for guiding antifungal therapy because of different *in vitro *susceptibility. The use of culture is limited by its modest sensitivity, long turnaround times, and the potential for both, misidentification based on morphologic characteristics and contamination producing false positive results. While histopathology can prove invasive fungal infections by the demonstration of fungal elements in tissue specimens, genus or species level identification due to morphologic characteristics is limited. Therefore, results from histopathology often are not useful for guiding antifungal therapy. In addition, results from non invasive experimental diagnostic tests (e.g. serologic assays or PCR from blood or BAL) may be difficult to interpret in the absence of a sensitive and accurate gold standard. Therefore, the lack of a sensitive gold standard for identifying the etiologic agents of IFI limits optimal patient care and the evaluation of new diagnostic tests from clinical samples.

Formalin fixed, paraffin embedded (FFPE) tissue specimens from patients with proven IFI deposited in pathology archives have been used to study the etiology of IFI using molecular methods. The yield of PCR and sequencing in identifying fungal DNA from FFPE tissue specimens in previous studies ranged from 60-68% [1,2]. Targeting the fungal ribosomal RNA by fluorescence in situ hybridisation (FISH) is an alternative molecular diagnostic approach that is not well studied in FFPE samples. FISH has the advantage of being very rapid and capable of detecting very few cells. In addition, it allows for the localisation of sequences to pathology in tissue samples. In this study, we sought to design FISH probes for the identification of fungal pathogens frequently encountered in human infections. Fluorescence-Microscopy (FM) with 4',6-Diamidin-2-phenylindole (DAPI) and FISH was used on FFPE tissue specimens from patients with proven IFI. The results of FM were compared with quantitative PCR targeting the fungal ribosomal RNA gene in order to assess the advantages and limitations of these two molecular approaches for the identification of the etiology of invasive fungal infections.

## Methods

### Design of FISH probes

For the construction of fungal probes targeting the fungal rRNA, an alignment containing the sequences of the 28S D1-D2 region of frequent fungal pathogens and the human rRNA gene was constructed using the clustal W algorithm in the Accelrys Gene software package (Accelrys, San Diego, CA). The alignment was scanned visually for potential probe sequences targeting medically important groups of fungi. Data on the accessibility of this region for FISH probes, as evaluated for *Saccharomyces cerevisiae*, were considered [3]. The number of mismatches of the designed probes to the sequence of medically important fungi was documented (Additional file 1, Table S1). The *in silico *specificity of potential probes was tested using Genbank and Probecheck (http://131.130.66.200/cgi-bin/probecheck/content.pl?id=home). All FISH-probes were synthesized by Eurofins Mwg/Operon (Huntsville, AL) and stored in sterile DNA-grade water at -20°C.

### Evaluation of FISH probes

The designed probes were further evaluated using cultivated fungal type strains fixed in formalin. Strains of *Aspergillus fumigatus *(ATCC MYA-1163), *Aspergillus terreus *(ATCC 10070), *Penicillium chrysogenum *(ATCC 10108), *Paecilomyces variotii *(ATCC 10865), *Candida albicans *(ATCC 90028), *Candida tropicalis *(clinical isolate), *Issatchenkia orientalis *(ATCC 14243), *Scedosporium prolificans *(ATCC 90470), *Fusarium. solani *(ATCC 56480), *Cunninghamella bertholletiae *(ATCC 42115), *Mucor racemosus *(ATCC 42647) and *Absidia corymbifera *(ATCC 14058) were used. Fungi were grown for 12-48 h in Sabauroud-Glucose broth at room temperature under constant agitation. When fungal elements became visible by eye, the broth was centrifuged and the supernatant was removed. The pelleted fungi were washed with PBS (phosphate buffered saline), centrifuged and PBS was removed. Fungi were fixed in formalin (35%; 1:1 with PBS) for 24 h, washed in PBS once and stored at 4°C in a 1:1 mixture of PBS and 96% ethanol until use.

For the evaluation of the FISH probes, the formalin fixed fungi were placed on polylysine-coated slides and air-dried. Prior to the application of the hybridisation buffer and probes, the slides were heated on a hot plate for 30 min at 100°C in order to increase binding of fungi to the slides. Hybridisation buffer consisted of 5× SET (0.75 M NaCl, 5 mM EDTA, 0.1 M Tris [pH 7.8]), 10% dextran, 0.2% BSA, 0.1 mg/ml polyadenosine, 20 μg/ml salmon testes DNA and 0.02% SDS. Fungi were covered with 20 μl of hybridisation buffer containing 40 ng of the FISH probes and 20 ng of DAPI. Cover slips were placed and the slides were incubated at 52°C for 4 hours in a humid chamber. After incubation, cover slips were removed by immersion in 5× SET at 4°C and the slides were washed three 3 × 10 minutes in 0.2× SET buffer. Vectashield mounting solution (Vector laboratories, Burlingame, CA) and a cover slip was applied for epifluorescence microscopy.

### Fluorescence microscopy of fungi

A Nikon epifluorescence microscope and Metavue image analysis software (Molecular devices, Sunnyvale, CA) was used to generate digital images. The fluorescence intensity was checked visually using standardised exposure times (FITC: 1000 ms, Dapi: 20 ms, Cy3: 200 ms). Fungal elements were screened for staining with DAPI. Fluorescence of the designed probes was compared with the fluorescence signal obtained with positive and negative controls (Table 1). A positive hybridisation signal with rRNA probes was defined as a signal higher than that of a negative probe labelled with the corresponding fluorophore. Non-specific fluorescence was defined as fluorescence detected with a negative probe or a broad fluorescence outside the target window, e.g. in wavelengths for which no fluorophores have been used. All hybridisation experiments with the type strains were done in triplicate.

**Table 1:** Characteristics of FISH probes used for hybridisation studies

Name	Target	use	dye	Sequence (5'-3')	Tm (°C)
EUK 516	18S	pos	5'-Cy3	ACC AGA CTT GCC CTC C	59.3
Univ. SSU	18S	pos	3'-FAM	GAC GGG CGG CGG TGT GTA CAA	58.3
Non 338	n.a.	neg	3'-Cy3	ACT CCT ACG GGA GGC AGC	59.5
Prev	16S	neg	3'-FITC	CCA CAT GTT CCT CCG CTT GT	59.4
D223	28S	broad	5'-Cy3	CCA CCC ACT TAG AGC TGC	62,2
D223IO	28S	broad	5'-Cy3	CCA CCC GCT TGG AGC TG	55.8
Asp F	28S	Asp	5'-FITC	TGA CGG CCC GTT CCA G	61.8
Cand 317	28S	*C.a*.,*C.g*	5'-FITC	CAC GTA CTT TTT CAC TCT C	55.8

Pos: positive Control, neg: Negative Control, broad: Broad spectrum fungal, Asp: Aspergillus group, *C.a*.: *Candida albicans *group, *C.g*.: *C. glabrata*

### Tissue sections

Tissue samples from patients with proven invasive fungal infections were identified in tissue banks at the Fred Hutchinson Cancer Research Center (FHCRC) and the University Hospital Frankfurt (Main), Germany. The study was approved by the institutional review board of the FHCRC and the University Hospital Frankfurt. Tissue samples from 33 patients with proven invasive fungal infection were analysed. Patient characteristics such as age, sex and underlying illness were extracted from the patient charts and are summarised in Table 2. Information on antifungal therapy and outcome of the infection were not available.

**Table 2:** Clinical Characteristics of 33 patients with proven invasive fungal infection

Age (years)	58.5 Median	(Range:19-83)
Sex	female	12
	male	21
Underlying disease	Haem. Malignancy	23
	Organ Transplant	3
	AIDS	2
	other	5
Tissue	post mortem	20
	intravital	13

Other: lung emphysema, bowel perforation, Crohn's disease, Crest's syndrome, pancreatic cancer.

Sections of 5 μm were cut from FFPE tissue blocks. They were placed on negatively charged slides for conventional histology or hybridisation with FISH probes. In addition, aliquots of four cuts from each sample were placed in Eppendorf Biopur tubes for DNA extraction for PCR. The first and the last section from each block were stained by Gomori-Methenamine-Silver (GMS) and Periodic acid-Schiff stain (PAS) to confirm the presence of fungal elements in the tissue sample. Fungal elements were classified either as yeasts (budding yeast cells and/or pseudohyphae), septate hyphae with parallel walls and regular branching or broad non-septate hyphae with irregular branching suggestive for mucormycosis.

### DNA extraction for PCR

DNA extraction and PCR setup were performed in a laminar air flow hood to avoid contamination by ubiquitous fungi. Before DNA extraction, paraffin was removed from the tissue samples by incubation in 1000 μl octane as previously described [4]. Sterile, disposable pestles were used to grind the tissue in the octane containing tubes. After incubation for 5 minutes at room temperature, 100 μl methanol (100%) was added. After centrifugation (13000 rcf for 5 min), the supernatant was removed and the samples were vacuum dried for 60 minutes. The MasterPure™ yeast DNA Purification Kit (Epicentre^® ^Biotechnologies, Madison, WI) was used for DNA extraction from the deparaffinised tissue samples. Modifications were introduced from the original protocol as suggested by the manufacturer for the extraction of DNA from cultured fungi as described previously [5]. In short, 550 μl Yeast Cell Lysis solution was added to the tubes containing the deparaffinised, dried tissue sections. The content of the tubes was transferred to 2 ml sterile screw cap tubes loaded with silicon carbide sharps (BioSpec Products, Bartlesville, OK) of sizes 0.1 and 1 mm at a 1:1 ratio up to a volume equivalent to 250 μl. After homogenisation in a FastPrep^®^-24 System (MP Biomedicals, Solon, OH) at 5 m/s for 60 s, the samples were incubated for 3 hours at 90° and then placed on ice for 5 minutes. MPC Protein Precipitation Reagent ™ (325 μl) was added, the tubes were vortexed for 10 seconds and than centrifuged at 11000 rcf for 10 minutes. The supernatant was transferred to a new micro-centrifuge tube containing 750 μl isopropanol (100%) pre-cooled to -20°C, mixed by inversion and incubated for one hour at -20°C. The precipitated DNA was pelleted by centrifugation (11000 rcf for 10 min). The supernatant was removed and discarded. The pellet was washed with 70% ethanol (500 μl), vortexed and centrifuged (11000 rcf for 5 min). After removal of the supernatant, the pellet was vacuum dried for 60 minutes. The pellet was than resuspended in 75 μl of 0.1% Triton × (pre-warmed to 65°C) and incubated at room temperature for 5 min with periodic gentle vortexing. The extracted DNA was stored at -20°C overnight and then used for qPCR.

The extraction was repeated from additional sections of four samples. In two samples, inhibition of the PCR reaction was detected by the internal amplification control (IAC), in one sample to confirm a mixed infection, and to confirm a sequencing result not in accordance with a result from culture in one sample. With each extraction of six samples, 3 extraction controls consisting of water, for a total of 23, were processed in parallel to monitor for contamination during the extraction procedure.

### Quantitative PCR assays

Quantitative real time PCR was performed with an Applied Biosystems 7500™ real-time instrument and EvaGreen^® ^(Biotium Inc, Hayward, CA), a double stranded DNA binding florescent dye. Fungal DNA was amplified using two broadrange PCR assays targeting the ribosomal RNA genes. The ITS-2 assay (primers 5.8 forward and 28S-1 reverse) amplifies a 200-360 bp product including the variable ITS-2 region of the fungal rRNA operon. The 28S assay (primers 28S-10 forward and 28S-12 reverse) amplifies a 330-350 bp fragment of the 28S rRNA gene. Both assays have been shown to amplify DNA from a broad range of fungi in the presence of human DNA [6]. Each 50 μl PCR mixture contained 1 × PCR buffer A, 0.65 × Eva Green (Biotium Inc, Hayward, CA), 3 mM of MgCl_2_, 1.2 mM of GeneAmp dNTP blend (12.5 mM with dUTP), 2.2 U of AmpliTaq Gold^®^, 0.05 U AmpErase^® ^Uracil N-glycosylase (all from Applied Biosystems, Foster City, CA), 40 (28S) or 30 (ITS-2) pmol/μl of the forward and reverse primer, 0.002% Triton-X 100 and 100 ng/μl bovine serum albumin (BSA; Sigma).

To prevent contamination by fungal DNA, each PCR mastermix without additional water was filtered through a Microcon YM-100 centrifugal filter (Millipore Corp., Billerica, MA) with a molecular weight cutoff (MWCO) of 100 kDa at 650 rcf for 25 min and 1500 rcf for 5 min. Albumin was filtered accordingly. Water added to the mastermix was filtered with an Ultra-15 centrifugal filter unit with a MWCO of 30 KDa (Millipore Corp., Billerica, MA).

The PCR cycling conditions consisted of uracil N-glycosylase activation at 50° for 2 min, premelt at 95°C for 10 min and then 45 cycles of 95°C for 15 sec (melt), 55°C for 30 sec (annealing), 72°C for 40 sec (extension) followed by a dissociation step.

Standard curves for quantifying yeast and mould DNA were generated using genomic DNA from *A. fumigatus *(ATCC MYA-1163) and *C. albicans *(ATCC 90028) ranging from 1000 pg to 10 fg. Fungal PCR assays were run in duplicate on all samples and extraction controls. A positive PCR result required a positive signal in less than 41 cycles with a dissociation curve showing the same peak temperature in both runs.

Successful DNA extraction was confirmed in all samples and extraction controls by quantification of the human 18S rRNA gene by qPCR as previously described [5]. A standard curve was generated using human genomic DNA (Roche, Indianapolis, IN) ranging from 1 to 10000 pg.

The presence of PCR inhibitors was assessed in all samples and extraction controls using an exogenous internal amplification control (IAC). If inhibition was detected, as manifested by more than 2 cycle delay in the IAC threshold cycle, the samples were considered indeterminate [5]. In this case extraction was repeated from new sections of the same blocks.

### Sequencing and Identification of fungi

The amplicons of all fungal PCR positive samples and extraction controls were sequenced. Amplicons were purified using the ExoSap-IT^®^-kit (USB-products, Cleveland, OH). Sequencing was done using the primers 5.8 F and 28S-10F. Sequences were used for a BLAST search using an internal database and Genbank to identify the amplicons. For the identification of fungi at the species level, a similarity of > 97% was required. As sequence information for the amplified 28S region are limited in public databases, a lower threshold (> 95%) was accepted for preliminary identification.

### Fluorescence microscopy from FFPE tissue specimens

Paraffin was removed from tissue sections by dipping the slides into 99% octane for 15 seconds at room temperature. The sections were air dried and then bleached by immersing the slides in a fresh solution of 100 mM Tris hydrochloride (pH = 8) and 50 mM sodium borohydride for 1 hour. After being rinsed in distilled water, the slides were air dried. Next, the tissue samples were preheated to 100°C for 30 min on a hot plate. To each slide, 60 μl of hybridisation buffer containing 120 ng of probe and 60 ng of DAPI were added and cover slips were placed. The slides were incubated at 52°C for 6 h in a humid chamber. Hybridisation buffer, incubation temperatures, removal of coverslips, and washes were done as described above for formalin fixed fungi.

Digital images were generated as described above for cultivated fungi. Each experiment contained the tissue slides incubated with the fungal probes and both positive and negative control probes with the corresponding fluorophore. As a positive control for the correct performance of the hybridisation procedure, formalin fixed fungi (*A. terreus, C. tropicalis, P. variotii *and *C. bertholletiae*) were included with each probe used in the experiment. The slides were scanned visually for fungal elements, for DAPI staining and for areas with positive hybridisation of the rRNA probes within fungal elements. A positive FISH signal was defined as fluorescence in excess of that detected with the negative control FISH probes. Fungal elements were checked for a non-specific fluorescence signal outside of the window of the probes used. A negative sample was defined as showing fungal elements without a fluorescence signal obtained with rRNA probes higher than the negative controls.

### Statistics

Statistical analysis (Fishers exact test and correlation coefficient) was done using the JMP software package (SAS). All tests for significance were two sided, and P values of less than 0.05 were considered to indicate statistical significance.

## Results

**Figure 1:**
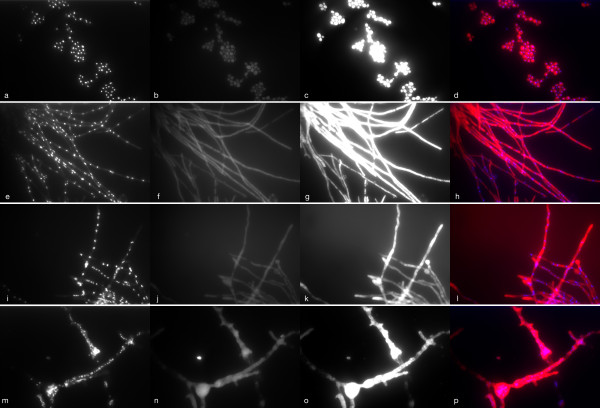
**Fluorescence microscopy of formalin fixed fungi (*C. tropicalis*: a-d, *A. terreus*: e-h, *P. variotii*: i-l, *C. bertholletiae*: m-p) with DAPI and the universal eukaryotic probe EUK 516 (labelled with Cy3)**. Images were collected in three wave-length channels: DAPI (first column), FITC (second column) representing non-specific fluorescence, Cy3 (third column). Last column represents a fusion image of the DAPI and Cy3-channels.

Fluorescence microscopy of cultivated fungi using DAPI showed a granular pattern of fluorescence reflecting integration of DAPI into fungal nuclear DNA (Figure 1 a, e, i, m). A varying degree of non-specific fluorescence was seen in all tested fungi (Figure 1 b, f, j, n). Hybridisation with positive control probes resulted in fluorescence of the fungal cytoplasm (Figure 1 c, g, k, o). Whereas yeast cells showed a homogenous fluorescence after hybridisation with the rRNA probes (Figure 1c), the intensity of fluorescence was more variable in different areas of the mould hyphae, suggesting inhomogeneous distribution of rRNA or differences in cell wall permeability along the length of hyphae (Figure 1 g, k, o).

One previously described and three newly designed probes were successfully applied to cultivated fungi (Table 1). Results of *in silico *analyses and hybridisation experiments with cultivated type strains are summarised in Table 3 and the **Table S1 **of the additional file 1. The probe D223 has been evaluated previously with various yeasts by Inacio [3]. The probe hybridises with the rRNA of a broad range of medically important fungi with the exception of *I. orientalis, Scedosporium *spp., *Fusarium *spp. and the mucorales. Probe D223 IO was designed to hybridise with rRNA of *I. orientalis*. Both probes were used together, labelled with Cy3, for the detection of frequent fungal pathogens such as *Candida *spp. and *Aspergillus *spp. in tissue sections. The probe Cand 317 was designed for the detection of causative agents of invasive candidiasis such as *C. albicans*, and *C. glabrata*. No hybridisation was detected with *I. orientalis*. In silico analysis suggests that hybridisation might be obtained with additional fungi such aus *S. cerevisiae, Pichia stipidis *and *Lodderomyces elongisporus *(Additional file 1, **Table S1**). The probe Asp F was designed to hybridise with rRNA of causative agents of invasive Aspergillosis. No hybridisation was detected with other Hyalohyphomycetes, yeasts or the mucorales. Fluorescence intensity comparable to the positive controls was obtained using these probes on cultivated fungal strains fixed with formalin. Due to a substantial heterogeneity of the D1-D2 region of the mucorales, no single probe could be generated that hybridized with all tested mucorales. Therefore, the positive control probes were used to determine the potential of FISH-probes to hybridise with hyphae of the mucorales in FFPE tissue sections.

**Table 3:** Hybridisation results obtained with FISH probes on type strains

number of mismatches (hybridisation detected)														
probe	*C.a*.	*C.g*.	*C.t*.	*I.o*.	*A.f*.	*A.t*.	*P.v*.	*S.p*.	*S.a*.	*F.s*.	*C.b*.	*M.r*.	*A.c*.	Human
D 223	0(+)	0(+)	0(+)	2(-)	1(+)	1(+)	1(+)	5(-)	4(nt)	3(-)	6(-)	4(-)	5(-)	3
D 223 IO	2(-)	2(-)	2(-)	0(+)	3(-)	3(nt)	3(nt)	4(-)	5(nt)	5(-)	6(-)	5(-)	4(-)	2
Cand 317	0(+)	0(+)	0(+)	2(-)	3(-)	3(-)	3(nt)	3(-)	3(nt)	1(-)	2(-)	3(-)	3(-)	5
Asp F	3(-)	3(-)	4(-)	4(-)	0(+)	0(+)	1(-)	5(-)	2(nt)	2(-)	3(-)	5(-)	5(-)	9
														

*C.a.: C. albicans, C.g.: C. glabrata, C.t*.: *C.tropicalis, I.o*.: *I. orientalis, A.f*.: *A. fumigatus, A.t.: A. terreus, P.v*.: *P. variotii, S.p*.: *S. prolificans, S.a*.: *S. apiospermum, F.s*.:*Fusarium solani, C.b*.: *C. bertholletiae, M.r.: M. racemosus, A.c*.: *A. corymbifera*. (+): hybridisation detected, (-): no hybridisation detected, (nt): not tested.

### PCR and sequencing from tissue specimens

Forty-two tissue samples from 35 patients were analyzed. As the IAC demonstrated significant inhibition in two samples from two patients, which was confirmed after repeated extractions, these samples were excluded from the analysis. Table 2 summarizes patient characteristics from 33 patients from whom the 40 tissue sections were obtained. Biopsies were taken from the lung (30), the gastrointestinal tract including the liver (6), paranasal sinuses (2), the skin (1) and a blood vessel wall with adjacent thrombus (1).

**Figure 2:**
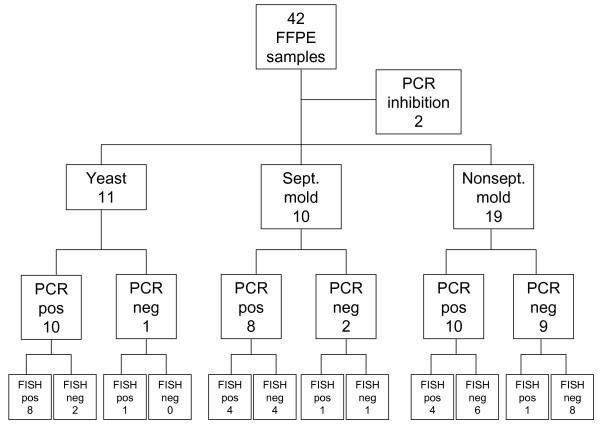
**Results from PCR and FISH in 42 samples from patients with proven invasive fungal infection**.

Fungal-PCR was positive in 28 of 40 samples (70%). PCR was most successful in samples with invasive yeast infection (10 of 11; 91%) and in samples with septate mould hyphae (8 of 10; 80%), but less successful in samples with non-septate mould hyphae (10 of 19; 53%) (Figure 2). The 28S rRNA gene PCR assay was positive in all 28 PCR positive samples, while the ITS-2-assay remained negative in 2 of 28 samples.

The amount of fungal DNA in positive samples ranged from 6 pg to 105 ng (Median: 480 pg) as determined by 28 S rRNA gene PCR and 14 pg to 357 ng (median: 2,25 ng) by ITS-2 PCR. In samples positive by both fungal PCR assays, the amount of fungal DNA was highly correlated (r^2 ^= 0.4; p = 0.001). Human DNA in clinical samples ranged between 3 pg and 1.1 μg per sample (median 109 ng). A cutoff > 940 pg of human DNA per sample was a predictor for a positive fungal DNA PCR (1/7 vs. 28/33; p = 0.001).

Sequencing of the fungal DNA-PCR amplicons identified 29 fungi in 28 samples. The sequences were homologous to sequences of *A. fumigatus *(8), *C. albicans *(6), *Rhizopus microsporus *(5), *C. tropicalis *(2), *A. corymbifera *(2), *I. orientalis, Cryptococcus neoformans, Scedosporium apiospermum, Rhizomucor miehei, C. bertholletiae *and *Rhizopus oryzae*. In 19 of 26 samples the same fungus was identified by sequencing of the amplicons from both PCR assays. In 6 samples from patients with mucormycosis, sequencing of the 28S amplicon did not allow for an identification of the amplicon due to poor sequence information in databases, while sequencing of the ITS-2 amplicon suggested *Rh. microsporus *(n = 5) and *Rh. oryzae *(n = 1) as the causative agent. However all sequences obtained by 28S-PCR showed homology to mucorales.

In one lung biopsy sample with negative culture results and histopathology suggesting budding yeast cells as well as hyphal elements, which were interpreted as pseudohyphae, sequencing of the amplicon obtained by the 28S-PCR identified *A. fumigatus*, while the ITS-2 identified *C. tropicalis*. The extraction was repeated and the dissociation curve of the ITS-2 PCR showed a dissociation curve that combines the typical temperature peaks seen for *A. fumigatus *and *C. tropicalis*, suggesting the presence of both fungi in the sample. Sequencing showed a mixture of two sequences that precluded identification. The 28S-PCR from the second extraction showed a dissociation curve characteristic for *A. fumigatus *and this was confirmed by sequencing.

In two samples only the 28 S rRNA gene PCR was positive. Sequencing of the amplicons suggested *C. bertholletiae *and *Rh. miehei *as the causative agents.

Culture results were available for 14 samples. Sequencing results were in accordance with culture results in 8 samples. In 5 cases of culture-proven mucormycosis both PCR-assays remained negative. In one sample a fungal isolate was grown from the biopsy and was identified as *Scopulariopsis brevicaulis*. Both fungal PCR-assays were positive. Sequencing of the 28S amplicon suggested an agent of the mucorales and the ITS-2 amplicon was identified as *Rh. microsporus *in accordance with the broad non-septate hyphae seen in histopathology.

A total of 23 extraction controls were processed together with the clinical samples. Of these, 3 were PCR-positive by the 28S rRNA gene PCR only. Sequencing did not reveal a single identifiable sequence in 1 of 3. In the other two samples, a sequence was obtained but identification was not possible in Genbank due to low similarity with deposited sequences, the closest match being the basidiomycetous yeast *P. cocos *(83% similarity). These sequences were not detected in any of the clinical sample.

### Fluorescence microscopy of tissue sections

**Figure 3:**
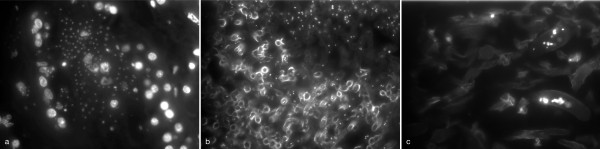
**Fluorescence Microscopy on tissue sections from patients with proven invasive fungal infection using DAPI**. Invasive Candidiasis showing fluorescence of nuclei of yeasts and host cells (a). Invasive mold infection showing limited integration of DAPI in a sample with septate-(b) and non-septate mold hyphae (c). In addition, intense fluorescence of fungal cell wall in parts of the septate mold hyphae (b).

FM of the tissue sections containing yeast cells, including short pseudohyphae, using DAPI showed a granular staining of the yeasts comparable with patterns seen in cultivated yeasts. In addition, nuclei of human cells were visualised by DAPI (Figure 3a). In contrast, in mould samples, DNA was stained by DAPI only in restricted parts of the hyphae (Figure 3 b, c). In addition, intense staining of the hyphal cell wall was seen in parts of the samples with septate mould hyphae (Figure 3b).

**Figure 4:**
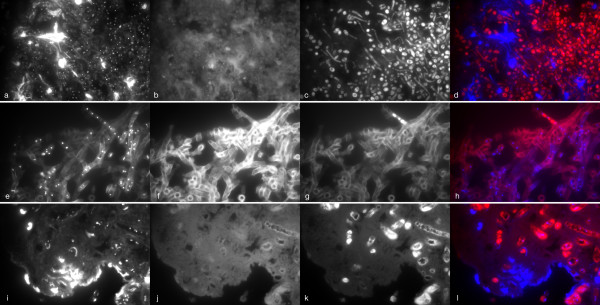
**Hybridisation of tissue sections with DAPI and a broad range fungal rRNA probe (D223 and D223IO labelled with Cy3: c-d and g-h) or eukaryotic universal probe (EUK 516 labeled with Cy3: k-l) with tissue sections from a patient with candidiasis (a-d), aspergillosis (e-h) and mucormycosis (i-l)**. DAPI-channel (first column), FITC-channel (second column), Cy-3 channel (third column), fusion picture of DAPI and Cy3-channels (last column).

**Figure 5:**
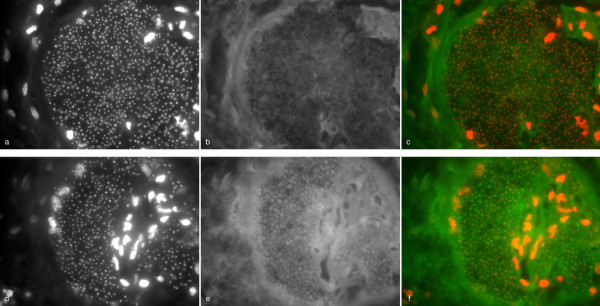
**Liver biopsy from a patient with hepatosplenic candidiasis without culture confirmation**. Sequencing of PCR amplicons revealed *C. tropicalis*. Probe Candida 317-FITC (d-f) shows hybridisation, in contrast to probe Asp-F-FITC (a-c). DAPI channel (first column), FITC-channel (second column), Fusion picture DAPI and FITC channels (last column). Note that there is substantial autofluorescence in the FITC channel.

Hybridisation with rRNA probes was documented in all yeast cells in positive specimens (Figure 4c). In contrast, hybridisation was restricted in most tissue sections with mold hyphae, ranging from a positive signal in restricted parts only (Figure 4h) to most of the hyphae (Figure 4k). A positive hybridisation signal for the rRNA broad-range fungal probe (D223 and D223 IO) was obtained in 9 of 11 tissue sections with yeasts, including one PCR negative sample (Figure 2). A positive signal with the FITC-labeled Candida 317 probe was seen in 5 samples with yeasts, confirming an infection due to *C. albicans, C. glabrata *or related organisms, in accordance with the results of sequencing in all 5 samples. No hybridisation signal was seen with the Aspergillus probe in all samples with yeasts. Figure 5 shows a liver biopsy from a patient with hepatosplenic candidiasis without culture confirmation. *C. tropicalis *was suggested as the etiologic agent by sequencing of both PCR-assays. In accordance with this, a positive hybridisation signal was obtained with the Cand 317 probe (Figure 5e) and not with the negative probes and the Asp F (Figure 5b) probe.

**Figure 6:**
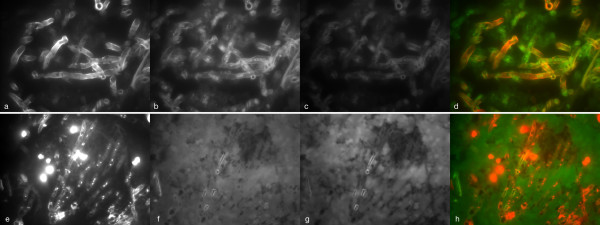
**Difficulties in the evaluation of hybridisation signals in samples with mould infection**. Autofluoresence of hyphae (a-d) or surrounding tissue (e-h) in two tissue specimens from patients with invasive aspergillosis. Hybridisation with DAPI and eukaryotic universal probe (univ-SSU labelled with FITC). DAPI channel (first column), FITC-channel (second column), Cy-3 channel (third column). Fusion picture DAPI and FITC channels (last column).

Interpretation of the hybridisation signal with rRNA-probes in samples with mold hyphae was challenging due to non-specific fluorescence of hyphal elements in one sample (Figure 6a-d). In 9 samples, intense non-specific fluorescence of the tissue surrounding the fungal elements impaired the assessment of hybridisation of rRNA probes to fungal elements, especially in the FITC-channel (Figure 6e-h). A positive hybridisation signal was obtained in 5 of 10 tissue sections with septate mould hyphae by the broadrange fungal probe. Hybridisation with the Asp F probe was positive in 3 of these 5 samples confirming invasive aspergillosis in accordance with results of sequencing that showed *A. fumigatus*. In one PCR negative sample, hybridisation with the broadrange fungal probe and Asp F suggests invasive aspergillosis. The candida 317 probe was negative in all samples with septate mold hyphae.

Hybridisation with the fungal elements was seen with the eukaryotic control probes in 5 of 19 samples with non-septate hyphae including one PCR-negative sample, suggesting that hybridisation with specific FISH probes might be obtained (Figure 2). The broad range fungal and the specific probes were negative in these samples.

All 7 samples with human DNA below 940 pg per sample, as measured by the human 18S-PCR, showed mold hyphae. In one of these samples fungal PCR identified *A. fumigatus*. FM showed abundant, DAPI positive hyphae, but no human cells. Microscopy showed necrotic tissue without staining of human cell nuclei by DAPI and without rRNA probe staining of human cells.

DAPI in fungal elements was seen in 30/40 samples (10/11 yeast; 9/10 with septate mold, 11/19 non-septate mold hyphae). DAPI in fungal cells was a predictor for a positive fungal PCR (24/30 vs. 4/10; p = 0.04).

## Discussion

The sensitive and reliable identification of fungal pathogens causing IFI is a prerequisite for the optimal use of antifungal therapies and for understanding the results of experimental diagnostic tests such as PCR and serologic/antigen based assays from clinical specimens. Using molecular techniques on archival tissue samples is an attractive approach to identify the etiology of IFI.

We developed and evaluated four rRNA-FISH probes targeting fungal pathogens frequently found in human infections. We compared rRNA-FISH hybridisation results with two broad-range fungal rRNA gene PCR's on FFPE tissue specimens from patients with proven IFI. The evaluation of the rRNA-FISH approach suggests that FISH probes may be applied successfully to FFPE tissue specimens from patients with proven IFI in order to characterise the etiology of IFI. A positive hybridisation signal was regularly seen in samples from patients with invasive candidiasis, documenting the potential of FISH in the identification of yeasts in FFPE tissue specimens. This is in accordance with results from in situ hybridisation (ISH) studies using probes targeting the ribosomal RNA of yeasts. Hybridisation was observed in all 21 FFPE samples from patients with culture proven invasive candidiasis [7]. FISH probes identifying a number of yeasts that require different antifungal treatment have been designed and were successfully applied to blood culture isolates [8,9]. However, the performance of these probes with FFPE tissue specimens has not been studied. By the use of a single probe or a mixture of probes, clinical meaningful information might be obtained, such as the exclusion or documentation of resistant fungi such as *I. orientalis*. In addition, mixed infections, that have been described in 5-9% of immunocompromised patients with invasive candidiasis may be detected by FISH probes using different fluorophores [10,11]. Given that 20% of stem cell transplant patients are colonised with multiple yeasts, there is a potential for underestimation of mixed infections with conventional diagnostics such as blood culture or histopathology [12].

A positive hybridisation signal was obtained in 50% of samples with septate mold hyphae using a broadrange fungal FISH probe in this study. The evaluation of these samples was clearly more challenging for a number of reasons. First, hybridisation signals were restricted to limited segments of hyphae in most of the samples. In a study using ISH, Park et al described weak or absent ISH signals in the centre of aspergillomas and intense signals along the edge of aspergillomas and in samples from invasive aspergillosis. They suggest that this reflects viability of the organisms [13]. They obtained positive hybridisation signals in 85% of the samples. This is in accordance with another study of ISH using probes hybridising with groups of fungi such as *Aspergillus *spp., *Fusarium *spp. or *Pseudallescheria boydii *[14]. In contrast to our study, these investigators included only culture positive samples that may have a higher tissue burden of viable fungi. If results of rRNA based imaging findings differ between viable and non-viable fungal elements, as has been suggested for coccidioidomycosis, these information might be useful for a documentation of a response to antifungal therapy [15]. Second, another limitation that became apparent in samples with septate mold hyphae was non-specific fluorescence, defined by a fluorescent signal outside the window of the probes used. While non-specific fluorescence of mold hyphae was seen in one sample only and is easily detected by checking different channels of the fluorescent microscope, non-specific fluorescence of the surrounding, mostly necrotic tissue limited the interpretation of hybridisation results, especially when FITC-labelled probes were used.

Due to the heterogeneity of the 28 S D1-D2 region in the agents of mucormycosis, no probe hybridising with all tested agents of mucormycosis could be designed and applied to tissue samples. More conserved regions, such as the 18S rRNA gene might be needed to design a single probe or a mixture of probes for hybridisation with the mucorales. In order to explore the potential of FISH to stain hyphae of the mucorales, the universal eukaryotic probes were used. A positive signal was obtained in 26% of the samples. In a study by Hayden using ISH on FFPE tissue samples, the interpretation of ISH results was most difficult in mucormycosis due to a weak hybridisation signal compared with a high degree of background signal [7]. The restriction of DAPI and rRNA-FISH positivity to limited hyphal parts in our study suggests that this is due to a low metabolic activity in most of the mycelium of the mucorales found in tissue. Further studies, including animal models might be useful to understand the patterns seen in FM of tissue specimens, especially to describe the influence of antifungal therapy.

Using two broad range fungal PCR assays allowed for the identification of important fungal pathogens such as *Candida *spp., *Aspergillus *spp., *Scedosporium *spp. and the agents of mucormycosis from 70% of FFPE tissue samples. This compares favourably with results from a previous study that detected fungi from 60% of FFPE tissue samples using two nested PCR assays targeting the 18S rRNA gene of the mucorales and the mitochondrial DNA of *Aspergillus *spp.. Beside the potential for contamination due to the nested PCR step, these assays do not allow for the identification of clinically important pathogens such as *Candida *spp., *Fusarium *spp. and *Scedosporium *spp., limiting their use in the identification of the etiology of IFI [1]. In another study, using the ITS-1 region of the fungal ribosomal RNA as the molecular target, Lau et al. were able to correctly identify a broad range of fungal pathogens as compared to culture results. However, although they used 10 sections of 10 μm for DNA extraction, they only identified fungal pathogens in 55% of FFPE tissue samples, suggesting a possibility for improving DNA extraction from FFPE specimens [2].

Both of our assays were designed to detect fungal DNA in an excess of human DNA without amplification of human DNA. While highly conserved regions as primer binding sites were avoided to prevent amplification of human DNA, less conserved regions as primer-binding sites may explain the lack of detection of certain mucorales by the ITS-2 assay. In contrast, the 28S assay amplified a broad range of fungi including some mucorales not detected by the ITS-2 assay. Amplification of fungal DNA in some extraction controls was noted with the 28S assay only. While this did not adversely affect the identification of fungi in clinical samples, it represents a limitation of broadrange fungal PCR assays. The identification of some mucorales was limited from the amplified sequences with this assay. While the ITS-2 assay suggested *Rh. microsporus *and *Rh. oryzae*, in two samples, sequencing of the 28S amplicon showed homology with sequences of mucorales below the chosen cutoff. The deposition of additional fungal sequence information of this area may further improve the usefulness of this assay. Consistent identification by sequencing of both amplicons in accordance with culture results suggests a high specificity of species identification with these assays. While a sensitive amplification was observed from samples with yeasts and septate mould hyphae, samples from mucormycosis were less sensitive. This is in accordance with previous studies that showed negative PCR results in 40% of the samples from patients with mucormycosis. In addition 60% of PCR negative samples in the study of Lau et al. were from patients with mucormycosis. This diagnostic conundrum requires further study.

The results from this study highlight several key points. First, the quantification of human DNA was a good predictor for positive fungal PCR assays. This may be caused by either a failure of DNA extraction or that non-viable tissue may have lower levels of fungal DNA. However, a low amount of human DNA may not always reflect extraction failure as suggested by one sample with positive fungal PCR that showed only hyphae in FM. Second, the positive FM using DAPI was a predictor for positive fungal PCR assays. Given the finding that only limited parts of hyphal elements may contain DNA, as visualized by DAPI, staining with DAPI may be used to guide which areas of samples might be most useful for fungal PCR. Samples negative by fungal PCR but with positive DAPI staining in hyphal elements suggests that improvements in DNA-extraction might be achievable. Third, although PCR and sequencing appears to be more sensitive in detecting the etiology of IFI from FFPE tissue specimens, hybridisation techniques may add additional important information. FISH may allow for a characterisation of fungi in selected PCR negative samples due to a low DNA content or if inhibition of PCR is present. FISH might not be associated with a potential limitation of broadrange PCR assays such as the preferred amplification of some amplicons in mixed infections. While the prevalence of mixed infections is not clear, studies using molecular methods described mixed infections in 2-8% of fungal infections. Studies using hybridisation techniques or a combination of different PCR assays described a higher incidence than studies using a single PCR assay [1,2,14,16].

## Conclusions

Targeting the fungal rRNA by FISH has some utility for the rapid identification of fungal infections from FFPE tissue specimens. Samples negative by PCR but positive by FISH suggest that both techniques provide additional information. Interpretation of hybridisation results may be limited by non-specific fluorescence due to frequent tissue necrosis in invasive mold infections. While FISH may allow for a rapid detection of certain resistant organisms such as *I. orientalis *or *P. variotii*, broad-range fungal PCR with sequencing is more successful in identifying fungal pathogens in FFPE tissue samples, with even better performance possible by enhancing DNA recovery from tissue. Using molecular methods for the identification of fungal pathogens from banked FFPE samples may improve our understanding of the etiology of IFI, leading to optimised empiric antifungal therapies. Additionally, in patients with failing empiric antifungal therapies, the rapid identification of fungi by these molecular methods may facilitate rational guidance for antifungal therapy, leading to improved patient outcomes.

## Competing interests

DF, DK, and PK have intellectual property related to use of PCR for detection of fungal pathogens.

## Authors' contributions

VR helped to design the study, optimised the qPCR assays, performed data analysis and drafted the manuscript. PDK participated in the design of the study, assisted in the development, optimisation and implementation of the qPCR assays, and helped draft the manuscript. DLK assisted in the development, optimisation and implementation of the qPCR assays, and helped draft the manuscript. DM facilitated the identification of histology samples, was responsible for histologic analysis of samples, and critically reviewed the manuscript. EL facilitated the identification of histology samples and was responsible for histologic analysis of samples. DNF conceived the study, participated in the design of the study, assisted with data analyses, and helped to draft and review the manuscript. All authors read and approved the final manuscript except for EL who passed away during the preparation of the manuscript.

## Pre-publication history

The pre-publication history for this paper can be accessed here:

http://www.biomedcentral.com/1471-2334/11/202/prepub

## Supplementary Material

Additional file 1 **Number of mismatches of fungal probes for selected fungi**. Table contains the number of mismatches of the evaluated fungal probes with selected fungi. Click here for file
